# Arginine kinase from *Haemonchus contortus* decreased the proliferation and increased the apoptosis of goat PBMCs in vitro

**DOI:** 10.1186/s13071-017-2244-z

**Published:** 2017-06-26

**Authors:** Muhammad Ehsan, WenXiang Gao, Javaid Ali Gadahi, MingMin Lu, XinChao Liu, YuJian Wang, RuoFeng Yan, LiXin Xu, XiaoKai Song, XiangRui Li

**Affiliations:** 1 College of Veterinary Medicine, Nanjing Agricultural University, Nanjing, 210095 People’s Republic of China; 2 Department of Veterinary Parasitology, Sindh Agriculture University, Tandojam, Pakistan

**Keywords:** *Haemonchus contortus*, Arginine kinase, PBMC, Cytokines, Proliferation, Apoptosis

## Abstract

**Background:**

Arginine kinase (AK), an important member of phosphagen kinase family has been extensively studied in various vertebrates and invertebrates. Immunologically, AKs are important constituents of different body parts, involved in various biological and cellular functions, and considered as immune-modulator and effector for pro-inflammatory cytokines. However, immunoregulatory changes of host cells triggered by AK protein of *Haemonchus contortus*, a parasitic nematode of ruminants, are still unknown. The current study was focused on cloning and characterisation of Hc-AK, and its regulatory effects on cytokines level, cell migration, cell proliferation, nitric oxide production and apoptosis of goat peripheral blood mononuclear cells (PBMCs) were observed.

**Methods:**

The full-length sequence of the Hc-AK gene was amplified by reverse transcription-polymerase chain reaction (RT-PCR) and sub-cloned into the prokaryotic expression vector pET-32a. The biochemical characteristics of recombinant protein Hc-AK, which was purified by affinity chromatography, were performed based on the enzymatic assay. Binding of rHc-AK with PBMCs was confirmed by immunofluorescence assay (IFA). Immunohistochemical analysis was used to detect localisation of Hc-AK within adult worms sections. The immunoregulatory effects of rHc-AK on cytokine secretions, cell proliferation, cell migration, nitric oxide production and apoptosis were determined by co-incubation of rHc-AK with goat PBMCs.

**Results:**

The full-length ORF (1080 bp) of the Hc-AK gene was successfully cloned, and His-tagged AK protein was expressed in the *Escherichia coli* strain BL21. The recombinant protein of Hc-AK (rHc**-**AK**)** was about 58.5 kDa together with the fused vector protein of 18 kDa. The biochemical assay showed that the protein encoded by the Hc-ak exhibited enzymatic activity. Western blot analysis confirmed that the rHc**-**AK was recognised by the sera from rat (rat-antiHc-AK). The IFA results showed that rHc-AK could bind on the surface of goat PBMCs. Immunohistochemically, Hc-AK was localised at the inner and outer membrane as well as in the gut region of adult worms. The binding of rHc**-**AK to host cells increased the levels of IL-4, IL-10, IL-17, IFN-γ, nitric oxide (NO) production and cell apoptosis of goat PBMCs, whereas, TGF-β1 levels, cell proliferation and PBMCs migration were significantly decreased in a dose dependent manner.

**Conclusions:**

Our findings suggested that rHc-AK is an important excretory and secretory (ES) protein involved in host immune responses and exhibit distinct immunomodulatory properties during interaction with goat PBMCs.

**Electronic supplementary material:**

The online version of this article (doi:10.1186/s13071-017-2244-z) contains supplementary material, which is available to authorized users.

## Background

*Haemonchus contortus* is a blood sucking abomasal nematode parasite of ruminants, causing a wide range of morbidity and mortality in livestock, including substantial losses such as anaemia, loss of body weight and growth [1, 2]. As there is a lack of an effective vaccine, control of the disease caused by *H. contortus* mainly relies on anthelmintics; however, anthelmintic resistance in *H. contortus* has become a severe problem around the world [3, 4]. Therefore, there is an urgent need to develop new drugs and vaccines, which is built on our deep understanding of the biology of this parasite at the molecular level.

Arginine kinase (AK), being a highly conserved member of the phosphagen kinase (PKs) family, has been studied extensively with a high degree of sequence similarity among various invertebrate species including *H. contortus* [5], *Trypanosoma cruzi* [6], *Caenorhabditis elegans* [7], *Heterodera glycines* [8], *Toxocara canis* and *Ascaris lumbricoides* [9] and proteobacteria [10]. In invertebrates, AK catalyses the reversible phosphorylation of arginine by MgATP to form phosphoarginine and MgADP [11]. Co-substrate of AKs and nitric oxide synthase (NOS), L-arginine has been considered to cause immunomodulation through nitric oxide (NO) synthesis via different biochemical pathways and decreased the level of pro-inflammatory cytokines [12]. It has been found that expression of AK in the yeast *Saccharomyces cerevisiae* resulted in resistance against pH variation and cellular energy stress [13] and that over-expression of AK in *T. cruzi* increased parasite survival under the pH and nutritional stress [14]. It is noteworthy that, AK from *Crassostrea gigas*, after induction with LPS, inhibited ATP hydrolytic activity, led to the up-regulation of extracellular ATP stimulation, which induced discrete cellular responses such as, pore formation in the plasma membrane, cytokines production and cell apoptosis [15, 16]. In addition, the induced expression levels of *Chlamys farreri* AK and NO concentration after LPS stimulation indicated that AK played an important role in immunomodulation during invading pathogens [17].

Previously, AKs were identified as ESPs from various insects and marine species, including cockroaches, lobster, shrimps, crabs, moth and mite. So far, AK was also reported as one of the constituents of ESPs in some nematodes, like *Teladorsagia circumcincta* [18], *Anisakis simplex* [19] and *Heligmosomoides polygyrus* [20]. Until now, only one study has reported on the biochemical characteristics of AK from *H. contortus* [21]. However, the functions of AKs of nematodes, especially in immune regulations by interacting with host PBMCs are still unclear. In this study, the *H. contortus* AK gene (Hc-AK) was cloned, characterised and the recombinant protein (rHc-AK) expressed in *Escherichia coli* used to evaluate the immune regulatory role on the goat PBMCs.

## Methods

### Animals

Chinese local crossbred goats (3–6 months old) from the research and teaching flock at Nanjing Agricultural University were kept indoors. Hay and whole shelled corn and water were provided ad libitum. To maintain a helminth free environment, the goats were dewormed at 2 weeks intervals with the levamisole (8 mg/kg body weight) orally to eliminate naturally acquired nematode infection. According to parasitological techniques, microscopic examination of the faecal sample for helminth infection was checked twice per week. Helminth free goats were used during the whole study period.

Sprague-Dawley (SD) rats (body weight 150–160 g) were purchased from the Experimental Animal Center of Jiangsu, PR China (Qualified Certificate: SCXK 2008–0004) and were kept in a microbe free environment with sterilised food and water.

### Parasite collection

*Haemonchus contortus* adult worms (strain Nanjing 2005) was maintained in 1-year-old helminth-free goats. Goats were orally infected with ~10,000 L3 larvae of *H. contortus*. To check for persistance of the parasite infection, eggs in faecal samples were examined microscopically on a weekly basis. Then the goats were slaughtered, and adult worms were collected from the abomasum of donor goats 28 days post-infection and were stored in liquid nitrogen until needed.

### PBMCs isolation and culture

Heparinized peripheral venous blood samples from dewormed healthy goats were collected and cultured as the procedure described by Yanming et al. [22]. PBMCs were separated by standard Ficoll-hypaque (GE Healthcare, Munich, USA) gradient centrifugation method [23]. After washing twice with Ca^2+^/Mg^2+^-free PBS (pH 7.4), the trypan blue exclusion test conducted for cell viability was more than 95% in all experiments. PBMCs were adjusted to the required density in cell culture medium (RPMI 1640 or DMEM), containing 10% heat-inactivated fetal bovine serum (FBS), 100 U/ml penicillin and 100 mg/ml streptomycin (GIBCO, Paisley, UK). For functional analysis, PBMCs were cultured in 24-well flat-bottomed culture plates (Costar, Cambridge, MA, USA) with varying concentrations of rHc-AK at 37 °C in 5% CO_2_.

### RNA isolation and construction of cDNA from *H. contortus*

Total RNA was extracted from adult worms of *H. contortus*, collected from donor goats. The RNA was isolated under RNase-free condition using Trizol (Invitrogen, Shanghai, China) according to the manufacturer’s instructions. Briefly, the worms were minced and homogenised for 30 min in pre-chilled pestle and mortar containing 1 ml Trizol. Then 200 μl of trichloromethane was added to the mixture and centrifuged at 10,000× *g* for 15 min at 4 °C. The supernatant was precipitated by addition of 0.25 volume of isopropyl alcohol per 1 ml of Trizol and incubated at -20 °C for 30 min. RNA was pelleted at 10,000× *g* at 4 °C for 10 min. Pellet was dried and washed 70% ethanol and then suspended in DEPC water. The cDNA was synthesised by reverse transcription reaction using cDNA Kit (Takara Biotechnology, Dalian, China) according to manufacturer’s instructions. The reaction was carried out in the presence of 3.5 μl dNTP (10 mM) mixture and 1.5 μl oligo (dT) primer. The reaction was then run at two different temperatures at 70 °C for 10 min and 42 °C for 5 min and then cold on ice for 2 min. The final concentration was adjusted and stored at -20 °C for further use.

### PCR amplification, cloning and expression of Hc-AK gene

For amplification, specific primers Hc-AK-F: 5′-GGA TCC ATG TCT GTT CCT CCG-3′ and Hc-AK-R: 5′-GAA TTC TCA AGC CTT CTT CTC CAG T-3′ were designed from *H. contortus* Arginine kinase complete CDS: (GenBank: JX422018.1) using Primer Premier 5.0 software. The ORF of Hc-AK was amplified from cDNA synthesised from *H. contortus* adult worms by reverse transcription-polymerase chain reaction (RT-PCR) with the restriction enzyme-anchored (underlined) *BamH* I and *EcoR* I, respectively. After PCR amplification the products were confirmed by 1% agarose gel electrophoresis and then purified by using E.Z.N.A. Gel Extraction Kit (Omega Bio-tech, Norcross, GA, USA), according to manufacturer’s instructions. The final volume of 50 μl PCR reaction was comprised of 2 μl cDNA, 1.0 U *Taq* DNA polymerase (TaKaRa Biotech, Dalian, China), 3.0 mM MgCl_2_, 400 μM dNTP mixtures, 50 μM 10× LA PCR buffer (Mg^2+^ Free), and 400 nM of each primer. The PCR cycling conditions were as follows: 94 °C for 5 min followed by 35 cycles of 94 °C (45 s), 55 °C (45 s), 72 °C (1.20 min), and then a final extension at 72 °C for 10 min.

Amplified PCR product was then cloned into a pMD19-T vector (TaKaRa Biotechnology) and transformed into *E. coli* (DH_5_α) competent cells (Invitrogen Bio-tech). The recombinant pMD19-T-Hc-AK clone was identified by PCR amplification and endonuclease digestion. The target fragments were purified and cloned in frame with the digested prokaryotic expression vector pET-32a (+) (Novagen, Shanghai, China) to generate the recombinant plasmid pET-32a (+)-Hc-AK. The recombinant plasmid was sequenced (Invitrogen Bio-tech) to confirm the correct insertion of Hc-AK gene in the accurate reading frame. The nucleotide sequence data inserted into the recombinant plasmid was analysed by sequence analysis with available sequences in GenBank databases using bioinformatics search tool (BLAST) (http://www.ncbi.nlm.nih.gov/BLAST/).

### Bioinformatics analysis and phylogenetic tree

The sequence identity of Hc-AK to that of known AK sequences available on National Center for Biotechnology Information (NCBI) was analysed by BLASTp and BLASTx (https://blast.ncbi.nlm.nih.gov/Blast.cgi). Then the amino acids sequences from different nematode species were aligned and compared using CLUSTALW1.8. The phylogenetic tree was constructed using the Neighbor-Joining method and visualised using the Molecular Evolutionary Genetics Analysis (MEGA) 6.0 program [24]. The protein sequence was used to predict N-terminal signal peptides (http://www.cbs.dtu.dk/services/SignalP/), GPI modification Site Prediction (http://mendel.imp.ac.at/sat/gpi/gpi_server.html), T cell motifs (DNAstar (EditSeq, Protean), B cell epitopes (http://tools.immuneepitope.org/bcell/) as well as membrane protein prediction http://www.cbs.dtu.dk/services/TMHMM/ by using bioinformatics approaches available on the internet.

### Expression of *H. contortus* AK protein

The recombinant plasmid pET-32a (+)-Hc-AK was transferred into competent cells *E. coli* BL21 (DE3) in Luria Bertini (LB) medium containing ampicillin (100 μg/ml), and the protein expression was induced by addition of 1 mM Isopropyl-b-D-thiogalactopyranoside (IPTG; Sigma-Aldrich, Shanghai, China) to the culture after the OD_600_ of the culture reached 0.6 at 37 °C. The cells were cultured for 6 h at 37 °C after the addition of 1 mM IPTG and collected by centrifugation. The pellet was lysed using lysozyme (10 μg/ml) (Sigma-Aldrich) followed by sonication, and then cell lysates were analysed by 12% (*w*/*v*) sodium dodecyl sulfate-polyacrylamide gel electrophoresis (SDS-PAGE). The recombinant proteins were purified by Ni^2+^-nitrilotriacetic acid (Ni-NTA) column (GE Healthcare, USA) according to the manufacturer’s instructions. An elution buffer (300 mM NaCl, 40 mM NaH_2_PO_4_, pH 8.0) containing 400 mM of imidazole, was utilised to wash the His-tagged proteins. The purity of the rHc-AK protein was determined by 12% SDS-PAGE followed by Coomassie blue staining. The concentration of recombinant proteins was determined according to the Bradford procedure [25], using bovine serum albumin (BSA) as a standard and then stored at -20 °C for functional analysis. The fusion protein pET-32a with the 109aa Trx•Tag™ thioredoxin protein and six histidines was obtained by induction of *E. coli* BL21 transformed with pET-32a (+) plasmid.

### Production of antibodies against recombinant protein

To generate polyclonal antibodies, about 0.5 mg of the purified rHc-AK protein was mixed with Freund’s complete adjuvant as a 1:1 mixture and injected subcutaneously into Sprague-Dawley (SD) rats (Qualified Certificate: SCXK 2008–0004; Experimental Animal Center of Jiangsu, PR China) at multiple places. After 2 weeks, the rats received booster doses with protein and Freund’s incomplete (1:1) mixture thrice at one-week intervals. Finally, the serum containing antibodies against rHc-AK was collected and stored until used. Sera collected before protein injection was used as negative.

### Western blot analysis of rHc-AK

Specific reactivity of antibodies with rHc-AK was detected by immunoblot as described previously [26]. After resolving the purified rHc-AK protein on 12% SDS-PAGE, gel was transferred to polyvinylidene difluoride (PVDF) membrane (ImmobilonP, Millipore, Billerica, USA) using a semi-dry system (Novablot, Hoefer, USA) in transfer buffer (39 mM glycine, 48 mM Tris, 0.0375% SDS, 20% methanol) at 1.1 mA/cm^2^ for 1 h. Followed by blocking the free sites on the membrane with 5% (*w*/*v*) skimmed milk powder, the membrane was incubated with rat antisera as a primary antibody at 1: 300 dilutions in TBS/0.05% Tween 20 (TBST) at 4 °C overnight. Then the membrane was washed thrice with several changes of TBST and incubated with secondary antibody horseradish peroxidase (HRP)-conjugated goat anti-rat IgG (1: 500 dilutions) for 2 h. The membrane was washed, and immunoreaction as a chromogenic substrate was visualised with diaminobenzidine (DAB, Sigma-Aldrich) within 3–5 min.

### Enzyme assay for AK protein

The Universal Fluorimetric Kinase Assay kit (Sigma-Aldrich) was used to monitor ADP formation from the enzyme reaction according to the manufacturer’s protocol. The amount of ADP production which is directly proportion to the kinase activity was measured in a 50 μl of total reaction mixture, containing 20 μl of kinase reaction solution with serial concentrations of recombinant protein, 20 μl of ADP sensor buffer and 10 μl of ADP sensor solution, and incubated at room temperature for 30 min. Data were analysed by subtracting the fluorescence intensity value of the zero ADP controls for each data points. Fluorescence intensity was monitored by SPECTRAFLUOR (TECAN, Maennedorf, Switzerland) with the wavelength pair of 540–590 nm for emission and excitation respectively.

### Detection of rHc-AK binding to goat PBMC

Binding of recombinant protein (rHc-AK) to goat PBMC was detected by using IFA. In detail, freshly collected PBMCs (1 × 10^5^) were incubated with or without (control) rHc-AK for 2 h, at 37 °C and 5% CO_2_. PBMCs were allowed to settle down for 20 min on poly-L-lysine-treated glass slides and then fixed in 4% paraformaldehyde in PBS for 30 min at room temperature (RT). PBMCs were permeabilized with 1% TritonX-100 in TBS for 15 min, washed three times and blocked with 2% BSA in PBS for 1 h at 37 °C. Cells were then incubated with primary antibodies (1:100 dilutions), rat anti-rHc-AK- IgG or normal rat sera (as control) for 4 °C overnight. After three washes with PBS, slides were maintained in dark with secondary antibody goat anti-rat IgG (Beyotime, Shanghai, China) coupled with Cy3 (1:1000 dilutions) for 30 min, followed by 1.5 μM 2-(4-Amidinophenyl)-6-indolecarbamidine dihydrochloride (DAPI; Sigma, St. Louis, Missouri, USA) for 5–6 min. Then PBMCs were washed, covered with a coverslip, immersed in Anti-Fade Fluoromount solution (Beyotime Institute of Biotechnology, China) and examined at 100× magnification on a laser scanning confocal microscope (LSM710, Zeiss, Jena, Germany). The digital images were captured using the Zeiss microscope software package ZEN 2012 (Zeiss).

### Localisation of Hc-AK in adult *H. contortus* (male/female) worms

Immunohistochemical analysis was performed according to the method stated previously [27]. Briefly, freshly collected *H. contortus* adult worms, were fixed in 4% formaldehyde-0.2% glutaraldehyde in PBS for 45 min and then dipped in TISSUE-TeK® O.C.T. compound (SAKURA Finetek, Torrance, USA). After being snap frozen in liquid nitrogen, worms were cut into Cryostat sections of 10 μm thickness and washed with PBS. To prevent non-specific bindings, 10% normal goat serum in PBS was used for 1 h, and then sections were incubated with specific rat-anti-rHc-AK antiserum (1:100 dilutions) or normal rat serum (control) for 2 h at 37 °C. After subsequent washing step (10 min × 3) with PBS, the sections were incubated with secondary antibody coupled with Cy3, goat anti-rat IgG for 1 h at 37 °C. For DNA staining, the sections were subjected to DAPI (Sigma, St. Louis, Missouri, USA) for 5 min and washed three times with PBS. Finally, the specimens were immersed in the Fluoromount solution to prevent fading during microscopic examinations.

### Detection of cytokines level by enzyme-linked immunosorbent assay (ELISA)

To evaluate cytokines level in the supernatant of cultured PBMCs, 1.5 × 10^6^ cells were seeded into 24-well plates (1 ml/well). Cells were incubated with ConA (10 μg/ml) and series concentration of rHc-AK (5, 10, 20, 40 and 80 μg/ml), recombinant protein of pET32a and equal volume of control buffer (PBS) in RPMI 1640 culture medium (containing 100 U/ml penicillin, 100 μg/ml streptomycin, 2 mM L-glutamine, 10% FBS) at 37 °C with 5% CO_2_ for 24 h. Supernatants were collected by centrifugation at 200× *g* for 10 min, and the concentration of IL-4, IL-10, IL-17, TGF-β1 and IFN-γ were measured by commercially available goat ELISA kits (Jian Chen, Nanjing, China) according to manufacturer’s instructions. The cell viability was checked using the trypan blue exclusion test before and after 24 h incubation. Experiments were performed in triplicate.

### Cell proliferation assay

Cell proliferation assay was performed as stated previously [28]. Briefly, freshly isolated PBMCs (1 × 10^6^ cells/ml) were seeded into 96-well plates, activated with ConA (10 μg/ml) at same time with a serial concentrations of rHc-AK (5, 10, 20, 40 and 80 μg/ml), recombinant protein of pET32a and same volume of PBS (control buffer) and incubated at 37 °C with 5% CO_2_ for 72 h. According to the manufacturer’s instructions, 10 μl of cell counting kit-8 (CCK-8) reagent (Beyotime Biotechnology, Haimen, Jiangsu, China) was added to each well, 4 h before harvesting and the absorbance were measured at 450 nm (OD_450_) using a microplate reader (Bio-Rad Laboratories, Hercules, CA, USA). Cells with control buffer set as control and the OD_450_ in control groups were set as 100%. Cell proliferation index was calculated by the formula: OD_450_ rHc-AK /OD_450_ control. Experiments were conducted in triplicate.

### Cell migration assay

The migration assay was performed using a Millicell® insert with 8.0 μm pores (Merck-Millipore, USA) as described earlier [29] according to the manufacturer’s instructions. 200 μl cells (1.5 × 10^6^ cells/ml) with varying concentrations of rHc-AK (5, 10, 20, 40 and 80 μg/ml), recombinant protein of pET32a and same volume of PBS (control buffer) were seeded into the upper chamber and similarly, the lower chamber was filled with 1300 μl RPMI 1640 medium. After 2 h incubation, the cells migrated through the 8 μm pore size polycarbonate membrane into the lower chamber were determined by a Neubauer counting chamber. The difference between the mean values was calculated using ANOVA. Each experiment was performed in triplicate.

### Nitric oxide production assay

Freshly separated PBMCs (1 × 10^6^ cells/ml) were washed twice with PBS and poured in 96-well plates, containing DMEM medium. According to the Total Nitric Oxide Assay Kit (Beyotime Biotechnology), NO production was measured by intracellular nitrite by PBMCs using Griess assay [30]. Absorbance values of the coloured solution were measured using a plate reader (Bio-Rad Laboratories, Hercules, California, USA) at 540 nm (OD_540_), and converted to micromoles per litre (μmol/l) using a standard curve that was generated by addition of 0 to 80 μmol/l sodium nitrite to fresh culture media. The individual experiment was performed in triplicate.

### Cell apoptosis assay

Flow cytometer analysis was carried out as described [22]. PBMCs were cultured with or without different concentrations of rHc-AK and empty pET32a protein for 24 h. The cells were then washed twice with Ca^2+^/Mg^2+^-free PBS pH 7.4. PBMCs were re-suspended in binding buffer, and apoptosis assay was performed according to the manufacturer’s instructions of the Annexin V-FITC kit (Miltenyi Biotec, Bergisch Gladbach, Nordrhein-Westfalen, Germany). Annexin V-FITC was added to the cell suspension for 15 min in the dark at RT. The stained cells were analysed by flow cytometry (BD Biosciences, San Jose, California, USA) just after the addition of propidium iodide (PI, Sigma-Aldrich) to the cell suspension.

### Statistical analysis

The statistical analyses were performed by using the GraphPad Premier 6.0 software package (GraphPad Prism, San Diego, California, USA). Data are presented as mean ± SEM. The differences between groups were compared by one-way ANOVA, followed by a Tukey test and considered statistically significant at *P* < 0.05.

## Results

### Molecular cloning and sequence analysis of Hc-AK gene

The amplified PCR products of Hc-AK gene were obtained from *H. contortus* cDNA using a specific pair of primers, and a correct fragment size of 1080 bp was detected. The recovered PCR product was successfully cloned into a pMD19-T vector and confirmed by restriction enzyme digestion using *BamH* I*/ EcoR* I (Additional file 1: Figure S1). The fragment was purified, and the exact size was confirmed by sequencing. Then the cloned Hc-AK gene was inserted into the prokaryotic expression vector pET32a and confirmed by restriction enzyme digestion with *BamH* I/ *EcoR* I. The digestion of recombinant pET32a-Hc-AK produced a fragment of about 1080 bp which is equal to the molecular mass of Hc-AK. These results indicated that Hc-AK had been successfully inserted into the frame of pET32a vector.

The identity of nucleotide and amino acid sequences of Hc-AK by using BLASTx and BLASTp showed high sequences similarity to well-known nematode AKs available in the NCBI database (https://blast.ncbi.nlm.nih.gov/Blast.cgi). Multiple sequence alignment showed that all sequences were closely related with conserved domain residues, guanidino specificity (GS), actin binding sites, ADP binding sites as well as arginine binding sites, assumed that these residues are involved in the functional activity of AK protein. The arginine kinase specificity is dependent on GS region that is variable among different species and could mediate a lock-and-key mechanism (Additional file 1: Figure S2). Sequence comparisons showed that of the sequence of Hc-AK was highly similar to sequences from *H. contortus* (99%; GenBank: CDJ90032), *Caenorhabditis briggsae* (88%; (GenBank: XP_002645008), *Caenorhabditis brenneri* (88%; GenBank: EGT52941), *Caenorhabditis elegans* (87%; GenBank: NP_509217), *Heterodera glycines* (83%; GenBank: AAO49799), *Oesophagostomum dentatum* (92%; GenBank: KHJ89945), *Ancylostoma duodenale* (90%; GenBank: KIH65495), *Dictyocaulus viviparus* (94%; GenBank: KJH41917), *Vicugna pacos* (98%; GenBank: XP_006219889), *Necator americanus* (93%; GenBank: XP_013303820) (Additional file 1: Figure S3). No signal peptide, GPI anchor and transmembrane domain were found in the deduced protein, whereas, T and B cell motifs were detected in protein structure (Additional file 1: Figures S4-S6).

### Expression, purification and immunoblot analysis of rHc-AK

**Fig. 1:**
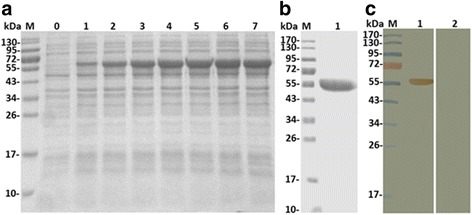
Expression, purification and Western blot analysis of rHc-AK. Lane M: standard protein molecular weight marker. **a** Expression of rHc-AK was induced with 1 mM IPTG. Lane 0: recombinant expression vector before induction; Lanes 1–7: protein expression at different time points. **b** Lane 1: purification of recombinant protein. **c** Lane 1: purified rHc-AK was electrophoresed and transferred to a membrane for Western blot analysis with rat anti-rHc-AK sera. Lane 2: membrane incubated with normal rat sera (as control)

In sonicated bacterial lysates, most of the rHc-AK protein was detected in the supernatant of the culture. The expression of the rHc-AK protein was detected after induction with 1 mM IPTG to the bacterial culture and samples were taken after each hour up to the 6 h to check expression at different time points (Fig. 1a). The rHc-AK protein was purified by chromatography with Ni-NTA super column, analysed on 12% SDS-PAGE and stained with Coomassie brilliant blue. A protein band of rHc-AK expressed product was about 58.5 kDa instead of the calculated molecular mass (40.5 kDa) due to extra pET-32a vector (Fig. 1). Western blot analysis showed that rHc-AK was detected by the sera Rat anti-rHc-AK, but could not be recognised by the sera from the rats before protein injection (control) (Fig. 1).

### Enzyme activity assay

**Fig. 2:**
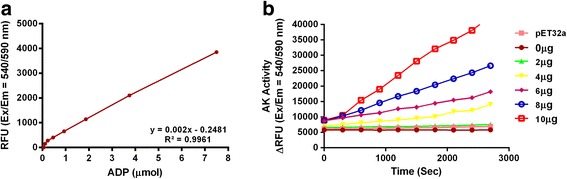
Enzyme activity of recombinant Hc-AK. **a** ADP calibration curve was generated for each experiment using kinase reaction buffer with serial dilutions (range: 0.05–30 μM) of ADP stock solution. **b** Kinase reaction was performed in an optimised ADP assay buffer system in the presence of different protein concentrations (0-10 μg) and monitored the fluorescence intensity (ƛ_ex_ = 540 nm/ƛ_em_ = 590 nm)

The results of functional activity assay of the recombinant Hc-AK are presented in Fig. 2. The amount of ADP produced in an optimised kinase buffer system coupled with serial concentrations (0, 2, 4, 6, 8 and 10 μg) of recombinant protein Hc-AK was evaluated, and results showed a remarkable augmentation in ADP generation with increased period and at dose dependent manner. A standard curve was generated to represent the amounts of ADP available in the reaction at the specified serial dilutions of ADP solutions. The control experiment using pET32a empty protein (10 μg/ml) and PBS showed no activity on enzyme reaction. These results confirmed that the observed enzyme activity in a kinase reaction mixture was due to the purified recombinant *H. contortus* AK protein (Fig. 2).

### Confirmation of rHc-AK binding to PBMCs

**Fig. 3:**
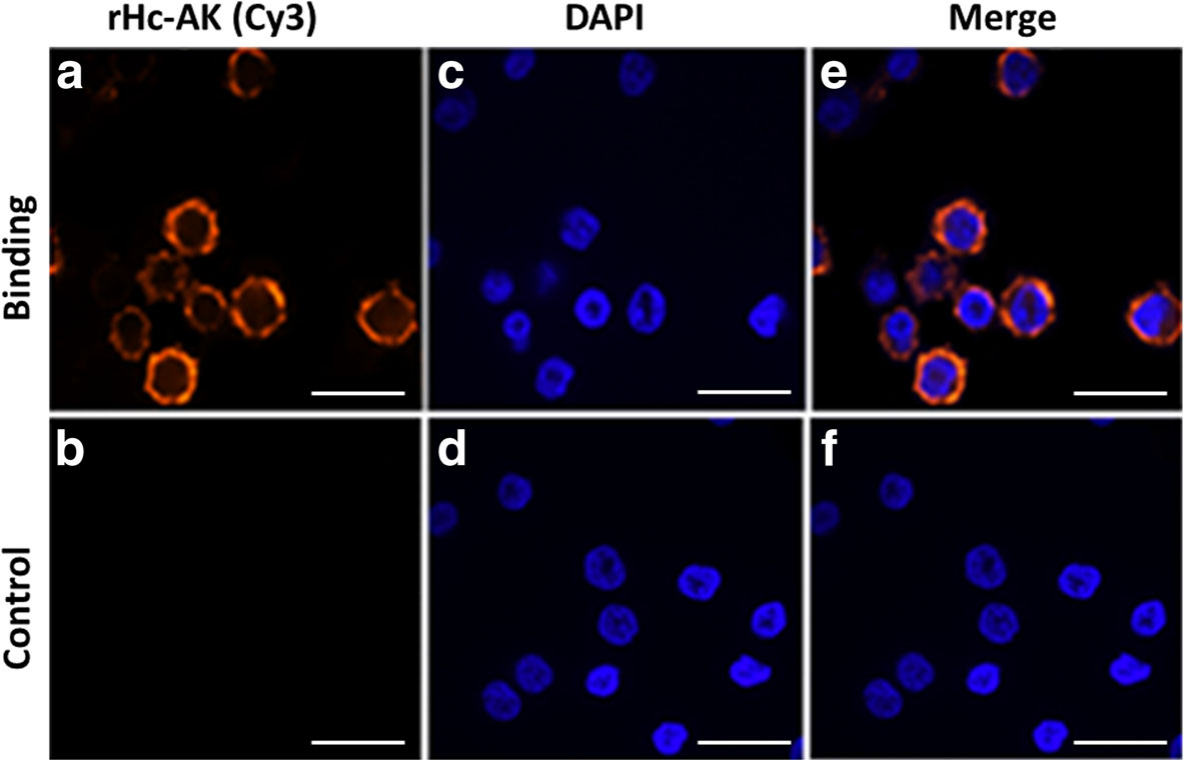
rHc-AK protein binding to goat PBMCs. Localisation was conducted by incubation of PBMC with rat anti-rHc-AK-O IgG or negative rat IgG (control). **a**, **b** Staining of the target protein (*red*) was utilised by the Cy3-conjugated secondary antibody. **c**, **d** Nuclei of corresponding cells were stained with DAPI (*blue*). **e**, **f** A merge overlaps of *red* and *blue* channels visualised by confocal microscopy. No *red* fluorescence was observed in control group. *Scale-bars*: 10 μm

The cultured goat PBMCs with or without rHc-AK were analysed by IFA using confocal microscopy (100× magnifications). As displayed in Fig. 3, the cells subjected to secondary antibody labelled with Cy3 showed in red colour, nuclei of the cells visualised by blue and combined image of protein binding were illustrated in merge colour of red and blue (Fig. 3 upper section). Whereas, there was no fluorescence observed in cells treated with control (Fig. 3 lower section). The dense concentration of red colour around the PBMCs indicated that rHc-AK could strongly bind to the cell surface.

### Immunohistochemical study of Hc-AK in adult *H. contortus* (m/f) worms

**Fig. 4:**
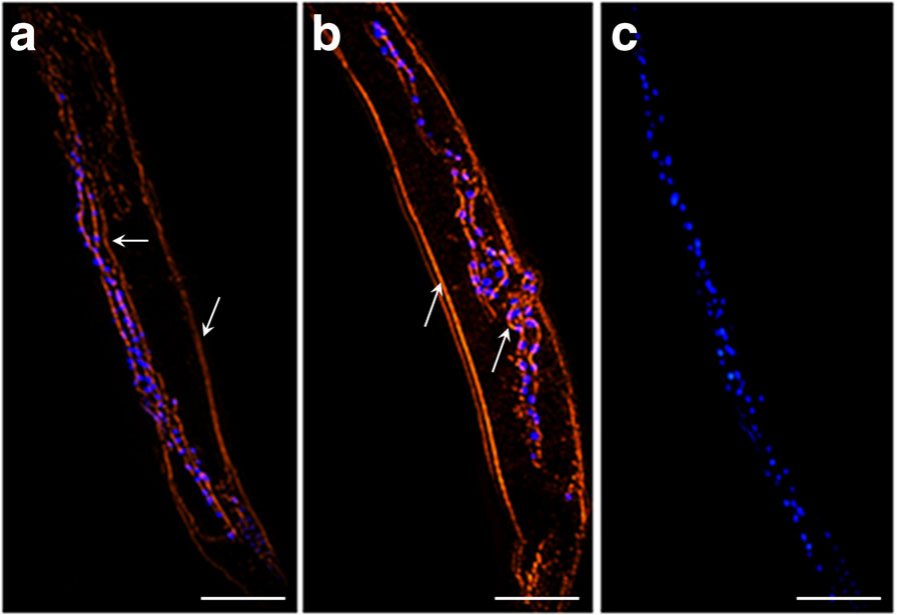
Localisation of Hc-AK in *H. contortus* adult worm by immunofluorescence assay. Nuclei were stained with DAPI (*blue*) and target protein with Cy3 (*red*). **a, b** Hc-AK localised at outer and inner membrane as well as the luminal surface of the male and female adult worm’s gut. **c** No fluorescence was observed in control. *Scale-bars*: 100 μm

A longitudinal section of a partial body length of adult *H. contortus* male and the female worm was shown in Fig. 4. Clusters of blue spots inside the body of worm indicated nuclei along the gut structure in both genders as well as a cross section of eggs in the female. The results showed that Hc-AK might be localised outer and inner surface of the membrane as well as in gut section (Fig. 4a, b). No protein labelling was observed in control section (Fig. 4c).

### Effect of the rHc-AK on individual cytokine secretion in PBMCs

**Fig. 5:**
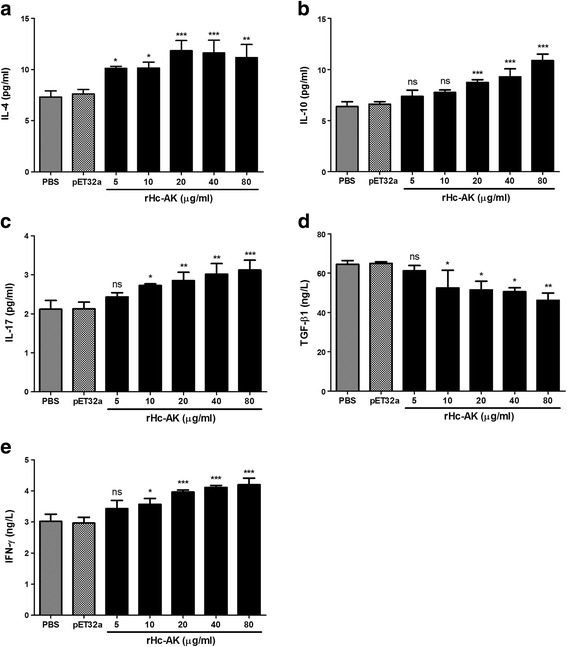
Effects of rHc-AK on multiple cytokines expression. PBMCs were stimulated with ConA (10 μg/ml) with PBS (control), pET32a (empty protein) and serial concentrations of rHc-AK for 72 h. Cytokines production in the supernatant of cell culture was quantified by ELISA. **a** IL-4. **b** IL-10. **c** IL-17. **d** TGF-β1. **e** IFN-γ. The data are representative of independent experiments triplicate in each (*P < 0.05, **P < 0.01, ***P < 0.001, ns, non-significant)

ELISA assay was performed to analyze the cytokines production by PBMCs treated with discrete concentration of rHc-AK (Fig. 5). It was noted that production of IL-4 (ANOVA, *F*_(6,20)_ = 9.025, *P* = 0.0001), IL-10 (ANOVA, *F*_(6,20)_ = 29.73, *P* = 0.0001), IL-17 (ANOVA, *F*_(6,20)_ = 12.84, *P* = 0.0001) and IFN-γ (ANOVA, *F*_(6,20)_ = 21.53, *P* = 0.0001) was significantly increased by the goat PBMCs incubated with different concentrations of rHc-AK (Fig. 5). Whilst, the level of TGF-β1 was prominently suppressed (ANOVA, *F*_(6,20)_ = 9.098, *P* = 0.0001) at dose dependent manner compared to the PBS (control) group and pET32a protein group (Fig. 5).

### PBMCs proliferation

**Fig. 6:**
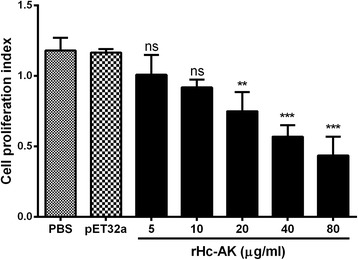
Impacts of different concentrations of rHc-AK on PBMCs proliferation. Cells were treated with control buffer, pET32a protein and serial concentrations of rHc-AK at 37 °C and 5% CO_2_. Proliferation test was conducted by CCK-8 incorporation after 72 h. Cell proliferation index was calculated considering the OD_450_ values in controls as 100%. The data are representative of triplicate experiments (**P < 0.01, ***P < 0.001)

The effect of rHc-AK on PBMCs multiplication was evaluated by incorporation of cell counting kit (CCK8). The analysis of results showed that rHc-AK produced significantly suppressive effect (ANOVA, *F*_(6,20)_ = 23.53, *P* < 0.0001) on goat PBMCs at 20 μg/ml, 40 μg/ml and 80 μg/ml protein concentrations (Fig. 6). Whereas, rHc-AK showed no significant difference on cell proliferation with 5 and 10 μg/ml (ANOVA, *F*_(6,20)_ = 23.53, *P* < 0.287) as compared to PBS (control) and pET32a empty protein group in interaction with PBMCs at dose-dependent manner (Fig. 6).

### Cell migration assay

**Fig. 7:**
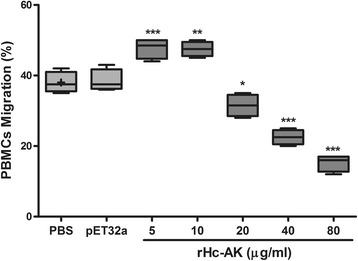
rHc-AK protein suppresses PBMCs migration. Cells were treated with control buffer, pET32a protein and serial concentrations of rHc-AK. The data are presented as box (50% of the values) and whiskers plot (Min and Max values), whereas median is designated by the horizontal bar, Results shown here is from one independent experiment (*n* = 4) and is representative of three independent experiments; (*P < 0.05, **P < 0.01, ***P < 0.001, ns, non-significant)

The effect of rHc-AK on the PBMCs migration was determined by a Millicell® insert (Corning, USA). It was noted that migration percentage was elevated (ANOVA, *F*_(6,20)_ = 65.06, *P* < 0.001) at the first step with 5 μg/ml (46.00 ± 2.082) and 10 μg/ml (48.33 ± 0.882) rHc-AK protein concentrations as compared to the PBS (control) group (38.33 ± 2.028) (Fig. 7). Furthermore, PBMCs migration percentage was decreased significantly (ANOVA, *F*_(6,20)_ = 65.06) at protein concentrations of 20 μg/ml (31.00 ± 2.082) (*P* < 0.013), 40 μg/ml (22.67 ± 1.453) and 80 μg/ml (14.00 ± 1.528) (*P* < 0.0001) as compared to the control group and empty vector protein group (38.67 ± 1.202) at dose-dependent manner (Fig. 7).

### Nitric oxide production

**Fig. 8:**
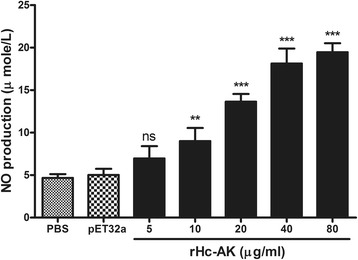
Influence of rHc-AK on intracellular nitric oxide production. PBMCs were treated with control buffer, pET32a protein and serial concentrations of rHc-AK at 37 °C and 5% CO_2_. The NO concentration in the PBMCs was measured by Griess assay. The data are presented as the mean ± SEM and representative of triplicate experiments (**P < 0.01, ***P < 0.001, ns, non-significant)

Total nitric oxide assay kit was used to evaluate the nitric oxide produced by PBMCs treated with varying concentration of rHc-AK and the same quantity of PBS (control) and pET32a protein. Results showed that nitric oxide production was significantly increased in cultured PBMCs at 20 μg/ml, 40 μg/ml and 80 μg/ml concentrations (ANOVA, *F*_(6,20)_ = 77.70, *P* < 0.0001) (Fig. 8). While, in the 10 μg/ml treatment group nitric oxide level was also increased but to a lesser extent compared with other groups (ANOVA, *F*_(6,20)_ = 77.70, *P* < 0.019). However, rHc-AK with 5 μg/ml concentration showed no effect (ANOVA, *F*_(6,20)_ = 77.70, *P* < 0.494) on nitric oxide production in cultured cells in vitro (Fig. 8).

### rHc-AK protein enhance apoptosis of goat PBMCs

**Fig. 9:**
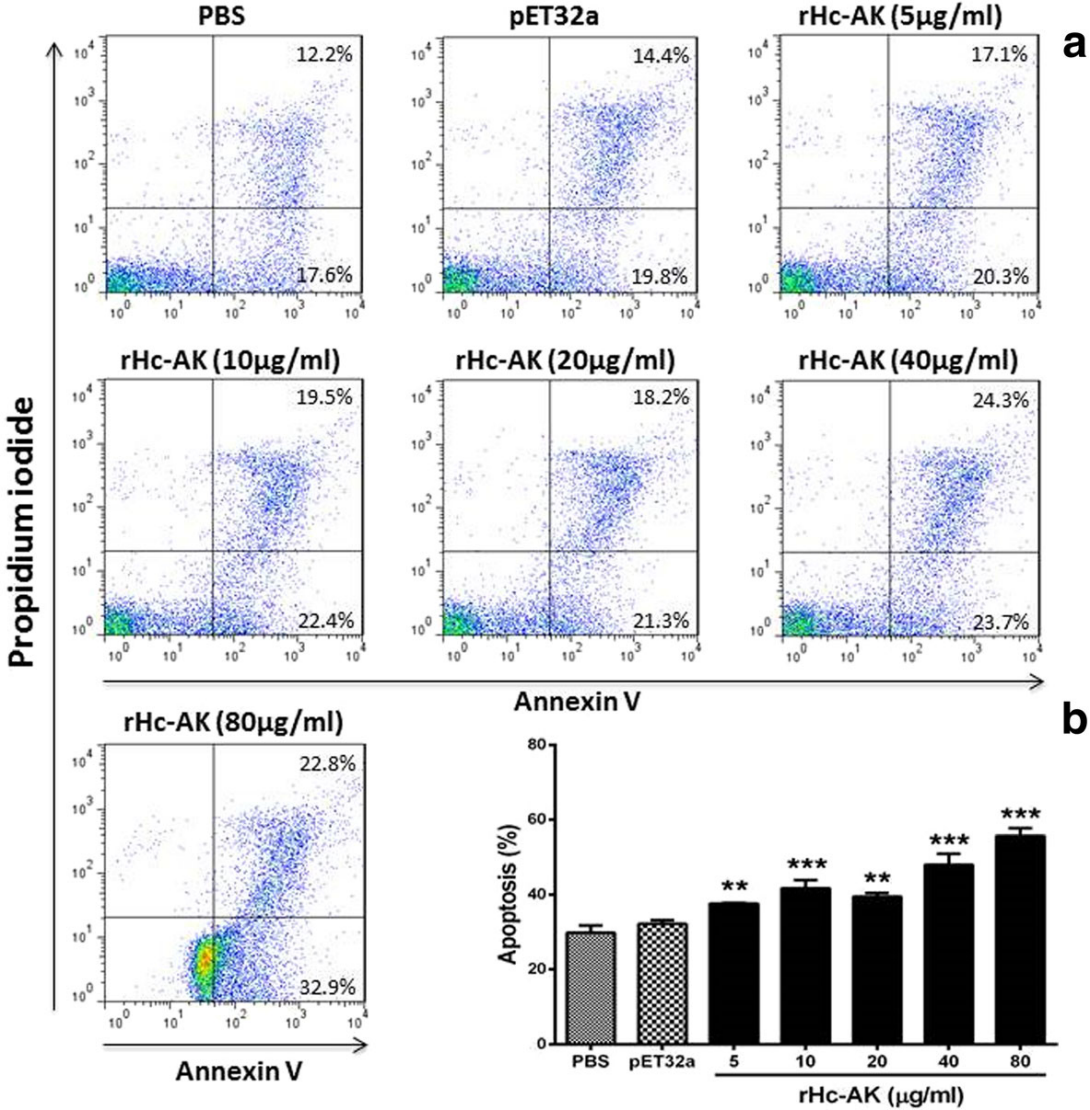
rHc-AK increases apoptosis of goat PBMCs at dose dependent manner. **a** Apoptosis of PBMCs was determined by staining with annexin V and PI followed by flow cytometry. The percentages of cells with different staining patterns are shown. **b** rHc-AK affects the apoptosis of goat PBMCs at different protein concentrations. The results are representative of triplicate experiments. Data are presented as the mean ± SEM (*n* = 3); an asterisk indicate treatment groups differ significantly (**P < 0.01) and highly significantly (***P < 0.001) from the control

To explore the impact of different concentration of rHc-AK on PBMCs apoptosis, a cell apoptosis assay was performed. The externalisation of membrane phosphatidylserine (PS) was used as a marker of cell apoptosis, and the positive DNA staining was used as an indicator of membrane leakage. The results showed that there was no significant change (ANOVA, *F*_(6,20)_ = 70.11, *P* < 0.137) between annexin V positive pET32a empty protein (30.20 ± 0.577) and control group (29.80 ± 1.155) (Fig. 9). Whereas, rHc-AK protein induced apoptosis of the goat PBMCs with protein concentration of 5 μg/ml and 20 μg/ml (ANOVA, *F*_(6,20)_ = 70.11, *P* < 0.017). However, rHc-AK dramatically augmented the apoptosis percentage (ANOVA, *F*_(6,20)_ = 70.11, *P* < 0.0001) at 10 μg/ml, 40 μg/ml and 80 μg/ml protein concentrations at dose-dependent manner as compared to the control group (Fig. 9).

## Discussion

Many excretory/secretory (ES) products of *H. contortus* including proteins belong to family phosphagen kinase (arginine kinase) were identified, that play a central role in host-parasite interaction by modulating host immune responses against parasitic infection, which are mostly Th2 responses prejudiced [31]. Interestingly, it was suggested that kinetic properties of AK played a potential part during immunomodulation [32] in response to the external stress or immune stimulations in invertebrates. PKs involved in cellular signalling transduction, metabolic processes, phosphorylation, DNA replication, cell proliferation, transcription, differentiation, cell-cycle progression, inflammation, apoptosis, and autophagy [33]. La Sala et al. [15] demonstrated that, the conversion of high-energy phosphate group or ATP molecules to arginine governed by AK required for metabolic and cellular activities that induce substantial mechanistic roles like cellular responses, alteration of the plasma membrane (pore formation), cytokine production and apoptosis. In the present study, a Fluorimetric Enzyme Assay showed an increased trend of the enzymatic activity of ADP production by the serial concentrations of rHc-AK at different time periods (Fig. 2). However, the interaction of Hc-AK with goat PBMCs and its immunoregulatory study has not been illustrated yet. We evaluated first in vitro functional analysis based on immunomodulation caused by *H. contortus* AK protein in interaction with goat PBMCs, which showed that the impact of rHc-AK changed trends of cytokine expression, proliferation, migration, nitric oxide production and apoptosis in different ways. This indicated that rHc-AK play a crucial role in the functional regulation of goat PBMCs.

All AKs are structurally monomers with a molecular size of approximately 40–45 kDa, which contained the similar functional domains, ATP-gua PtransN domain as specific for guanidine substrate domain (GS domain) and ATP-gua Ptrans domain responsible for ATP binding [34]. In this study, the alignment of deduced amino acid sequence of Hc-AK shared 80–99% similarity (Additional file 1: Figure S2), that contained GS region and ATP binding sites conserved in all nematodes AKs. The NJ phylogenic analysis showed that Hc-AK was closely related to other nematode species (Additional file 1: Figure S3). We cloned and characterised Hc-AK, with predicted molecular mass of 40.5 kDa, and specific antibodies were recognised by the rat sera experimentally infected with recombinant protein (Fig. 1). Together, sequence analysis and phylogenic relationship suggested that Hc-AK being a member of PK family, possess similar physical and functional characteristics of its structure.

It was demonstrated by immunohistochemically, that AK was localised in the cytosol and also found in actin-containing regions, in Z-line and A band region of tubular muscle [35]. Yu et al. [36] suggested that localisation of AK in growth cones served as direct elaboration. Furthermore, immunologically AKs present in metabolically active body parts such as muscles, ovaries, uterus and intestines of some nematode parasites [37]. Parasite ESPs contains many proteins that can challenge the host immune system either by modulation or suppression of their functions and this mechanism is governed by the interaction of these ES proteins with receptors on the surface of the host cell in shape of receptor-ligand complexes [38]. In this study, the immunofluorescence assay determined that rHc-AK could bind on the surface of goat PBMCs (Fig. 3). The adult *H. contortus* male and female sections were exposed immunohistochemically for Hc-AK localisation, and we found that protein was expressed in outer and inner membrane section as well as the gut region of the parasite (Fig. 4). However, the effects of Hc-AK on the subpopulation of PBMCs need to be further investigated.

Type 1 (Th1), type 2 (Th2) and inflammatory responses associated with secretion of various cytokines play an important role in the inhibition of parasitic infection especially *H. contortus* [39]. In this study IL-4, IL-10, IL-17, IFN-γ and TGF-β1 cytokines were selected to represent Th2, anti-inflammatory, pro-inflammatory, Th1 and Treg cytokine, respectively. To date, there is no information available regarding the effects of Hc-AK on cytokines secretions by goat PBMCs. Xing et al. [40] demonstrated that AK from Der f 20 induced IL-4 secretions in cultured splenocytes, leading to the Th2 cell differentiation and allergic responses. In our recent study, an ES antigen rHcES-24 was found to increase the IL4 production in PBMCs [41]. In this study, we suggested that rHc-AK could initiate the Th2 responses by production of IL-4 in goat PBMCs (Fig. 5) and help to eliminate the parasite. T regulatory cells (T_reg_) and its typical cytokine IL-10 mainly exert their suppressive effects on the development of Th2 allergic responses, which are significantly susceptible to parasitic infections. In this investigation, rHc-AK significantly increased the secretion of IL-10 in goat PBMCs and could facilitate worm infection by inducing Treg cells to produce immunosuppressive cytokine IL-10. Th17 effectors cells, capable of producing IL-17 cytokine, are associated with inflammatory responses and pathogenesis of various parasites [42, 43]. In accordance with our previous studies of HcESPs, rHcFTT-2 and rHcES-24 on cytokines production [41, 44, 45], it was suggested that, rHc-AK protein also participated in negative development of Th2 responses on IL-10 production (Fig. 5) and also involved in inflammatory reactions, favorable for parasite pathogenesis and survival by producing IL-17 in goat PBMCs.

Th1 cells produce pro-inflammatory cytokine, IFN-γ regulates cellular immunity against infection. Cope et al. [46], and associated with pathogen recognition, suppression of cell proliferation, immunomodulation and signal transduction in response to other cytokines [47]. Coomes et al. reported that IFN-γ could inhibit the development of Th2 immune responses [48]. In our previous study, the rHcFTT-2 protein increased the IFN-γ production by goat PBMCs in vitro [45]. In current research IFN-γ production was increased in goat PBMCs incubated with different rHc-AK concentrations (Fig. 5). TGF-β1 is a multifunctional cytokine that potentially regulates different immunomodulatory activities and biological processes, pro-inflammatory responses and immunosuppressive properties [49, 50]. In this study, we demonstrated that rHc-AK decreased the level of TGF-β1 in goat PBMCs (Fig. 5). The decreased level of TGF-β1 might be due to the antagonistic mechanism of IFN-γ, and the actual phenomena in impairment of Th1, Th2 mediated immune responses during host-parasite interaction need to be further investigation.

Complex regulatory activities, such as cell activation, cytokine secretion and cell cycling lead to cell proliferation. It was demonstrated that arginine being an enzymatic partner of the kinase with appropriate concentration could inhibit the proliferation and tumour growth in cancer cells based on metabolic pathway [51, 52]. Loke et al. [53] reported that immune responses in helminths infection could be altered by regulating the proliferation of immune cells. In our previous research, we found a suppressive regulation of HcESPs, rHcFTT-2 and rHcES-24 on the cell proliferation in vitro [41, 44, 45]. Similarly, in the current study, rHc-AK could significantly inhibit the cell proliferation in goat PBMCs (Fig. 6). This immunosuppressive effect created by AK or arginine itself needs to be further researched.

It was suggested that helminths actively initiate immune cells (eosinophil; lymphocytes) stimulation and trafficking to the infected site to combat pathogens [54], and these movement and effective functions are driven by chemokines or cytokines involved in this regulation [55]. In this study, cell migration was increased at 5 and 10 μg/ml of rHc-AK, and then it gradually declined with increased levels of AK protein (Fig. 7). The real mechanism in this fluctuation and factors involved in this suppressive regulation of cells trafficking needs further study.

Nitric oxide (NO) is a ubiquitous signalling molecule, recognised as the versatile player with numerous immunoregulatory and cytotoxic activities in the immune system. Previously, it was noted that NO had been involved in nonspecific defence mechanism against varieties of parasites including *H. contortus* [56]. Jiang et al. [57] stated that AK with LPS activation could activate the immune responses via a change in NO concentration. L-arginine as co-substrate of AK and iNOS also considered to play a role in the immune response of vertebrates [12] and has been involved in immunomodulation on NO production through metabolism of L-arginine by NOS [58]. In our study, a constant increase in NO production (Fig. 8) indicated that rHc-AK involved in the immunomodulatory regulation of NO on goat PBMCs.

Apoptosis is a naturally occurring phenomenon, probably regulated by two major routes, known as extrinsic (death receptors) and intrinsic (mitochondrial) pathways [59]. The extrinsic pathway is initiated by extracellular ligands that bind and activate death receptors on the cell membrane, while the intrinsic pathway can be activated by cell damage or during specific developmental stages [60]. The apoptosis is considered as immunoregulator of host immune responses induced by parasitic molecular and cellular mechanisms [61]. Previous studies determined that many molecules of galectin family and their binding partners involved in cell apoptosis [38, 62]. In our study goat, PBMCs showed a significantly high degree of rHc-AK-induced apoptosis (Fig. 9). Therefore, our results suggested that decrease trend of proliferation along with induction of apoptosis might be an immunosuppressive strategy of *H. contortus* should be investigated further.

## Conclusion

Our findings indicated that Hc-AK is a very important and active protein of HcESPs that might play important roles in the immune regulations. Our findings demonstrated that IL-4, IL-10, IL-17, IFN-γ, NO production and cell apoptosis were increased by Hc-AK. However, TGF-β1 level, PBMCs proliferation and PBMCs migration were decreased by the interaction of Hc-AK. These findings will not only contribute to understanding the functions of Hc-AK but might also help elucidate the general mechanisms involved in the immune responses and immune evasion by nematodes during host-parasite interactions.

## Additional file

Additional file 1: Figure S1. Cloning and expression of Hc-AK gene. The recombinant plasmid pMD19T-AK (**a**) and expression plasmid pET32a (+)-AK (**b**), were verified by restriction digestion with *BamH* I and *EcoR* I. **Figure S2.** Multiple sequence alignment of Hc-AK. Amino acid sequence of Hc-AK with that from other species, *H. contortus* (CDJ90032), *C. briggsae* (XP_002645008), *C. brenneri* (EGT52941), *C. elegans* (NP_509217), *H. glycines* (AAO49799), *O. dentatum* (KHJ89945), *A. duodenale* (KIH65495), *D. viviparus* (KJH41917), *V. pacos* (XP_006219889) and *N. americanus* (XP_013303820) using CLUSTAL W method and GeneDoc (http://www.psc.edu/biomed/genedoc/). **Figure S3.** Phylogenetic analysis for Hc-AK gene. A phylogenetic tree was constructed by neighbour -joining method to verify relationships between the amino acid of Hc-AK to that of other nematode species, using MEGA ver. 6.1 programme. **Figure S4.** N-terminal signal peptide prediction. The amino acid sequences of Hc-AK (NCBI accession numbers: JX422018.1 was used to predict N-terminal signal peptides by SignalP 4.1 Server. **Figure S5.** Membrane protein prediction by using TMHMM Server v.2.0. The amino acid sequences of Hc-AK (NCBI accession numbers: JX422018.1) was analysed to predict transmembrane structures using TMHMM Server v.2.0. There were no transmembrane domains predicted in this protein structure. http://www.cbs.dtu.dk/services/TMHMM/. **Figure S6.** Prediction of B and T cell epitopes. Protein sequence of Hc-AK (NCBI accession numbers: JX422018.1) was used for the prediction of the B cell and T cell epitopes, that revealed 16 peptides of B cell epitopes and 17 T cell epitopes. (DOCX 736 kb)

## Abbreviations

AK: Arginine kinase
BSA: Bovine serum albumin
Con A: Concanavalin A
Cy3: Cyanine dyes 3
DAB: Diaminobenzidine
DAPI: 2-(4-Amidinophenyl)-6-indolecarbamidine dihydrochloride
FBS: Fetal bovine serum
IFN-γ: Interferon-γ
IgG-HRP: Horseradish peroxidase labeled immunoglobulin G
IL-10: Interleukin-10
IL-17: Interleukin-17
IL-4: Interleukin-4
IPTG: Isopropyl-B-D-thiogalactopyranoside
LB: Luria Bertini medium
Ni-NTA: Ni^2+^-nitrilotriacetic acid
O.C.T: Optimal cutting temperature
PBMCs: Peripheral blood mononuclear cells
PBS: Phosphate buffered saline
PVDF: Polyvinylidene difluoride
rHco-gal-m/f: Recombinant galectins of male and female *Haemonchus contortus*
RPMI 1640: Roswell Park Memorial Institute 1640 culture media
SDS-PAGE: Sodium dodecyl sulfate polyacrylamide gel electrophoresis
TGF-β1: Transforming growth factor-β1
Th1: Helper T cell 1
Th2: Helper T cell 2

## References

[1] Schallig HD, van Leeuwen MA, Cornelissen AW (1997). Protective immunity induced by vaccination with two *Haemonchus contortus* excretory secretory proteins in sheep. Parasite Immunol.

[2] Muleke CI, Ruofeng Y, Lixin X, Yanming S, Xiangrui L (2006). Characterization of HC58cDNA, a putative cysteine protease from the parasite *Haemonchus contortus*. J Vet Sci.

[3] Jackson F, Coop RL (2000). The development of anthelmintic resistance in sheep nematodes. Parasitology.

[4] Wolstenholme AJ, Fairweather I, Prichard R, von Samson-Himmelstjerna G, Sangster NC (2004). Drug resistance in veterinary helminths. Trends Parasitol.

[5] Platzer EG, Thompson SN, Borchardt DB, Gamble HR (1995). High energy phosphate metabolites observed by NMR in infective larvae of *Haemonchus contortus*. J Parasitol.

[6] Pereira CA, Alonso GD, Paveto MC, Iribarren A, Cabanas ML, Torres HN (2000). *Trypanosoma cruzi* arginine kinase characterization and cloning. A novel energetic pathway in protozoan parasites. J Biol Chem.

[7] Uda K, Fujimoto N, Akiyama Y, Mizuta K, Tanaka K, Ellington WR (2006). Evolution of the arginine kinase gene family. Comp Biochem Phys D.

[8] Matthews BF, Macdonald MH, Thai VK, Tucker ML (2003). Molecular characterization of arginine kinases in the soybean cyst nematode (*Heterodera glycines*). J Nematol.

[9] Kulathunga DG, Wickramasinghe S, Rajapakse RP, Yatawara L, Jayaweera WR, Agatsuma T (2012). Immunolocalization of arginine kinase (AK) in *Toxocara canis*, *Toxocara vitulorum*, and *Ascaris lumbricoides*. Parasitol Res.

[10] Suzuki T, Soga S, Inoue M, Uda K (2013). Characterization and origin of bacterial arginine kinases. Int J Biol Macromol.

[11] Alonso GD, Pereira CA, Remedi MS, Paveto MC, Cochella L, Ivaldi MS (2001). Arginine kinase of the flagellate protozoa *Trypanosoma cruzi*. Regulation of its expression and catalytic activity. FEBS Lett.

[12] Kotlyar S, Weihrauch D, Paulsen RS, Towle DW (2000). Expression of arginine kinase enzymatic activity and mRNA in gills of the euryhaline crabs *Carcinus maenas* and *Callinectes sapidus*. J Exp Biol.

[13] Canonaco F, Schlattner U, Pruett PS, Wallimann T, Sauer U (2002). Functional expression of phosphagen kinase systems confers resistance to transient stresses in *Saccharomyces cerevisiae* by buffering the ATP pool. J Biol Chem.

[14] Fernandez P, Haouz A, Pereira CA, Aguilar C, Alzari PM (2007). The crystal structure of *Trypanosoma cruzi* arginine kinase. Proteins.

[15] La Sala A, Ferrari D, Corinti S, Cavani A, Di Virgilio F, Girolomoni G (2001). Extracellular ATP induces a distorted maturation of dendritic cells and inhibits their capacity to initiate Th1 responses. J Immunol.

[16] Borsellino G, Kleinewietfeld M, Di Mitri D, Sternjak A, Diamantini A, Giometto R (2007). Expression of ectonucleotidase CD39 by Foxp3 + Treg cells: hydrolysis of extracellular ATP and immune suppression. Blood.

[17] Shi X, Wang L, Zhou Z, Yang C, Gao Y, Wang L (2012). The arginine kinase in Zhikong scallop *Chlamys farreri* is involved in immunomodulation. Develop & Comp Immunol.

[18] Craig H, Wastling JM, Knox DP (2006). A preliminary proteomic survey of the *in vitro* excretory/secretory products of fourth-stage larval and adult *Teladorsagia circumcincta*. Parasitology.

[19] Faeste CK, Jonscher KR, Dooper MMWB, Egge-Jacobsen W, Moen A, Daschner A (2014). Characterisation of potential novel allergens in the fish parasite *Anisakis simplex*. EuPA Open Proteomics.

[20] Hewitson JP, Harcus Y, Murray J, van Agtmaal M, Filbey KJ, Grainger JR (2011). Proteomic analysis of secretory products from the model gastrointestinal nematode *Heligmosomoides polygyrus* reveals dominance of venom allergen-like (VAL) proteins. J Proteome.

[21] Umair S, Knight JS, Bland RJ, Simpson HV (2013). Molecular and biochemical characterisation of arginine kinases in *Haemonchus contortus* and *Teladorsagia circumcincta*. Exp Parasitol.

[22] Yanming S, Ruofeng Y, Muleke CI, Guangwei Z, Lixin X, Xiangrui L (2007). Vaccination of goats with recombinant galectin antigen induces partial protection against *Haemonchus contortus* infection. Parasite Immunol.

[23] Nicholson IC, Mavrangelos C, Fung K, Ayhan M, Levichkin I, Johnston A (2005). Characterisation of the protein composition of peripheral blood mononuclear cell microsomes by SDS-PAGE and mass spectrometry. J Immunol Methods.

[24] Tamura K, Stecher G, Peterson D, Filipski A, Kumar S (2013). MEGA6: molecular evolutionary genetics analysis version 6.0. Mol Biol Evol.

[25] Bradford MM (1976). A rapid and sensitive method for the quantitation of microgram quantities of protein utilizing the principle of protein-dye binding. Anal Biochem.

[26] Han K, Xu L, Yan R, Song X, Li X (2012). Molecular cloning, expression and characterization of enolase from adult *Haemonchus contortus*. Res Vet Sci.

[27] Wang W, Wang S, Zhang H, Yuan C, Yan R, Song X (2014). Galectin Hco-gal-m from *Haemonchus contortus* modulates goat monocytes and T cell function in different patterns. Parasit Vectors.

[28] Gadahi JA, Wang S, Bo G, Ehsan M, Yan R, Song X (2016). Proteomic analysis of the excretory and secretory proteins of *Haemonchus contortus* (HcESP) binding to goat PBMCs *in vivo* revealed stage-specific binding profiles. PLoS One.

[29] Taylor A, Verhagen J, Blaser K, Akdis M, Akdis CA (2006). Mechanisms of immune suppression by interleukin-10 and transforming growth factor-beta: the role of T regulatory cells. Immunology.

[30] Sun J, Zhang X, Broderick M, Fein H (2003). Measurement of nitric oxide production in biological systems by using Griess reaction assay. Sensors.

[31] Hewitson JP, Grainger JR, Maizels RM (2009). Helminth immunoregulation: the role of parasite secreted proteins in modulating host immunity. Mol Biochem Parasitol.

[32] Arockiaraj J, Vanaraja P, Easwvaran S, Singh A, Alinejaid T, Othman RY (2011). Gene profiling and characterization of arginine kinase-1 (MrAK-1) from freshwater giant prawn (*Macrobrachium rosenbergii*). Fish Shellfish Immunol.

[33] Ali AS, Ali S, El-Rayes BF, Philip PA, Sarkar FH (2009). Exploitation of protein kinase C: a useful target for cancer therapy. Cancer Treat Rev.

[34] Tada H, Suzuki T (2010). Cooperativity in the two-domain arginine kinase from the sea anemone *Anthopleura japonicus*. II. Evidence from site-directed mutagenesis studies. Int J Biol Macromol.

[35] Reddy S, Houmeida A, Benyamin Y, Roustan C (1992). Interaction *in vitro* of scallop muscle arginine kinase with filamentous actin. Eur J Biochem.

[36] Yu-mei EW, Esbensen P, Bentley D (1998). Arginine kinase expression and localization in growth cone migration. J Neurosci.

[37] Kenyon GL, Reed GH (1983). Creatine kinase: structure-activity relationships. Adv Enzymol Mol Biol.

[38] Sun Y, Yan R, Muleke CI, Zhao G (2007). Xu l, Li X. Recombinant galectins of *Haemonchus contortus* parasite induces apoptosis in the peripheral blood lymphocytes of goat. Int J Pept Res Ther.

[39] Shakya KP, Miller JE, Horohov DW (2009). A Th2 type of immune response is associated with increased resistance to *Haemonchus contortus* in naturally infected Gulf Coast native lambs. Vet Parasitol.

[40] Xing P, Yu H, Li M, Xiao X, Jiang C, Mo L (2015). Characterization of arginine kinase, anovel allergen of *Dermatophagoides farinae* (Der f 20). Am J Trans Res.

[41] Gadahi JA, Li B, Ehsan M, Wang S, Zhang Z, Wang Y (2016). Recombinant *Haemonchus contortus* 24 kDa excretory/secretory protein (rHcES-24) modulate the immune functions of goat PBMCs *in vitro*. Oncotarget.

[42] Larkin BM, Smith PM, Ponichtera HE, Shainheit MG, Rutitzky LI, Stadecker MJ (2012). Induction and regulation of pathogenic Th17 cell responses in schistosomiasis. Semin Immunopathol.

[43] Da Matta Guedes PM, Gutierrez FR, Maia FL, Milanezi CM, Silva GK, Pavanelli WR (2010). IL-17 produced during *Trypanosoma cruzi* infection plays a central role in regulating parasite-induced myocarditis. PLoS Neg Trop Dis.

[44] Gadahi JA, Yongqian B, Ehsan M, Zhang ZC, Wang S, Yan RF (2016). *Haemonchus contortus* excretory and secretory proteins (HcESPs) suppress functions of goat PBMCs *in vitro*. Oncotarget.

[45] Gadahi JA, Ehsan M, Wang S, Zhang Z, Wang Y, Yan R (2016). Recombinant protein of *Haemonchus contortus* 14-3-3 isoform 2 (rHcftt-2) decreased the production of IL-4 and suppressed the proliferation of goat PBMCs *in vitro*. Exp Parasitol.

[46] Cope A, Le Friec G, Cardone J, Kemper C (2011). The Th1 life cycle: molecular control of IFN-gamma to IL-10 switching. Trends Immunol.

[47] Schroder K, Hertzog PJ, Ravasi T, Hume DA (2004). Interferon-gamma: an overview of signals, mechanisms and functions. J Leukoc Biol.

[48] Coomes SM, Pelly VS, Kannan Y, Okoye IS, Czieso S, Entwistle LJ (2015). IFN-γ and IL-12 restrict Th2 responses during helminth/*Plasmodium* co-infection and promote IFN-γ from Th2 cells. PLoS Pathog.

[49] Li MO, Wan YY, Sanjabi S, Robertson AK, Flavell RA (2006). Transforming growth factor-beta regulation of immune responses. Annu Rev Immunol.

[50] Massague J (1990). The transforming growth factor-beta family. Annu Rev Cell Biol.

[51] Nanthakumaran S, Brown I, Heys SD, Schofield AC (2009). Inhibition of gastric cancer cell growth by arginine: molecular mechanisms of action. Clin Nutr.

[52] Caso G, McNurlan MA, McMillan ND, Eremin O, Garlick PJ (2004). Tumour cell growth in culture: dependence on arginine. Clin Sci.

[53] Loke P, MacDonald AS, Robb A, Maizels RM, Allen JE (2000). Alternatively activated macrophages induced by nematode infection inhibit proliferation via cell-to-cell contact. Eur J Immunol.

[54] McGovern KE, Wilson EH (2013). Role of chemokines and trafficking of immune cells in parasitic infections. Curr Immunol Rev.

[55] Klion AD, Nutman TB (2004). The role of eosinophils in host defense against helminth parasites. J Allergy Clin Immunol.

[56] James SL (1995). Role of nitric oxide in parasitic infections. Microbiol Rev.

[57] Jiang S, Jia Z, Chen H, Wang L, Song L (2016). The modulation of haemolymph arginine kinase on the extracellular ATP induced bactericidal immune responses in the Pacific oyster *Crassostrea gigas*. Fish Shellfish Immunol.

[58] Miller N, Saada R, Fishman S, Hurwitz I, Susswein AJ (2011). Neurons controlling “Aplysia” feeding inhibit themselves by continuous NO production. PLoS One.

[59] Steller H (1995). Mechanisms and genes of cellular suicide. Science.

[60] Sharon A, Finkelstein A, Shlezinger N, Hatam I (2009). Fungal apoptosis: function, genes and gene function. FEMS Microbiol Rev.

[61] Donskow-Schmelter K, Doligalska M (2005). Apoptosis, a protective mechanism for pathogens and their hosts. Wiad Parazytol.

[62] Li Y, Yuan C, Wang L, Lu M, Wang Y, Wen Y (2016). Transmembrane protein 147 (TMEM147): another partner protein of *Haemonchus contortus* galectin on the goat peripheral blood mononuclear cells (PBMC). Parasit Vectors.

