# Supplementary material for: Arginine kinase from Haemonchus contortus decreased the proliferation and increased the apoptosis of goat PBMCs in vitro
Source: Parasit Vectors. 2017 Jun 26;10:311. doi: 10.1186/s13071-017-2244-z (PMC5485575; doi:10.1186/s13071-017-2244-z)
Supplement: Additional file 1: Figure S1. — Cloning and expression of Hc-AK gene. The recombinant plasmid pMD19T-AK (a) and expression plasmid pET32a (+)-AK (b), were verified by restriction digestion with BamH I and EcoR I. Figure S2. Multiple sequence alignment of Hc-AK. Amino acid sequence of Hc-AK with that from other species, H. contortus (CDJ90032), C. briggsae (XP_002645008), C. brenneri (EGT52941), C. elegans (NP_509217), H. glycines (AAO49799), O. dentatum (KHJ89945), A. duodenale (KIH65495), D. viviparus (KJH41917), V. pacos (XP_006219889) and N. americanus (XP_013303820) using CLUSTAL W method and GeneDoc (http://www.psc.edu/biomed/genedoc/). Figure S3. Phylogenetic analysis for Hc-AK gene. A phylogenetic tree was constructed by neighbour -joining method to verify relationships between the amino acid of Hc-AK to that of other nematode species, using MEGA ver. 6.1 programme. Figure S4. N-terminal signal peptide prediction. The amino acid sequences of Hc-AK (NCBI accession numbers: JX422018.1 was used to predict N-terminal signal peptides by SignalP 4.1 Server. Figure S5. Membrane protein prediction by using TMHMM Server v.2.0. The amino acid sequences of Hc-AK (NCBI accession numbers: JX422018.1) was analysed to predict transmembrane structures using TMHMM Server v.2.0. There were no transmembrane domains predicted in this protein structure. http://www.cbs.dtu.dk/services/TMHMM/. Figure S6. Prediction of B and T cell epitopes. Protein sequence of Hc-AK (NCBI accession numbers: JX422018.1) was used for the prediction of the B cell and T cell epitopes, that revealed 16 peptides of B cell epitopes and 17 T cell epitopes. (DOCX 736 kb) [file 13071_2017_2244_MOESM1_ESM.docx]

**Additional file:**

**Figure S1:** Cloning and expression of Hc-AK gene. The recombinant plasmid pMD19T-AK (**a**) and expression plasmid pET32a (+)-AK (**b**), were verified by restriction digestion with *BamH* I and *EcoR* I and the products were resolved by 1 % agarose gel electrophoresis. M: DNA molecular marker.

**
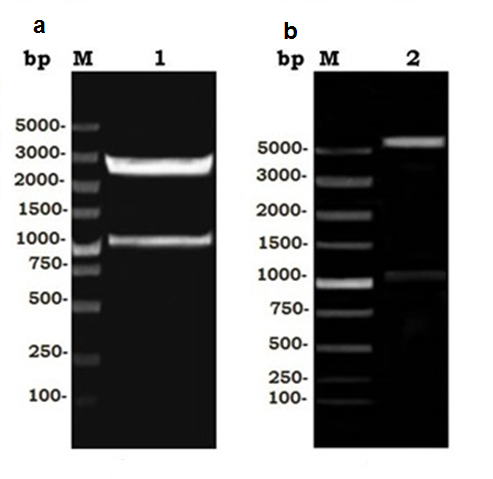
**

**Figure S2:** Multiple sequence alignment of Hc-AK. Amino acid sequence of Hc-AK with that from other species, *H. contortus* (CDJ90032), *C. briggsae* (XP_002645008), *C. brenneri* (EGT52941), *C. elegans* (NP_509217), *H. glycines* (AAO49799), *O. dentatum* (KHJ89945), *A. duodenale* (KIH65495), *D. viviparus* (KJH41917), *V. pacos* (XP_006219889) and *N. americanus* (XP_013303820) using CLUSTAL W method and GeneDoc (http://www.psc.edu/biomed/ genedoc/). Residues in blue colour indicate highly conserved, Guanidino specificity (GS) region is shown in a yellow box, actin binding sites in red box, Residues marked by green boxes are assumed to be involved in AK functions. The symbol * indicates arginine binding sites and # represents ADP binding sites.


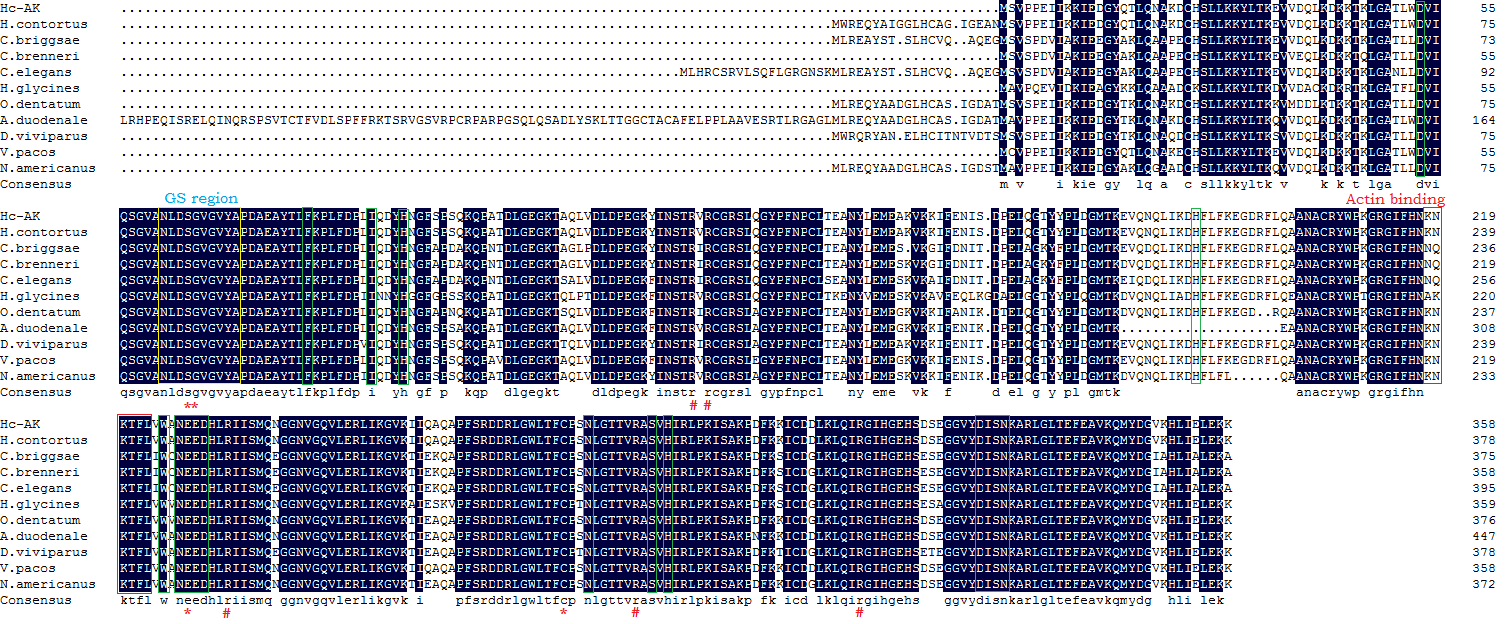


**Figure S3:** Phylogenetic analysis for Hc-AK gene. A phylogenetic tree was constructed by neighbour -joining method to verify relationships between amino acid of Hc-AK to that of other nematode species, using MEGA ver. 6.1 program. Values on the branches represent the bootstrap for 1000 replicates.


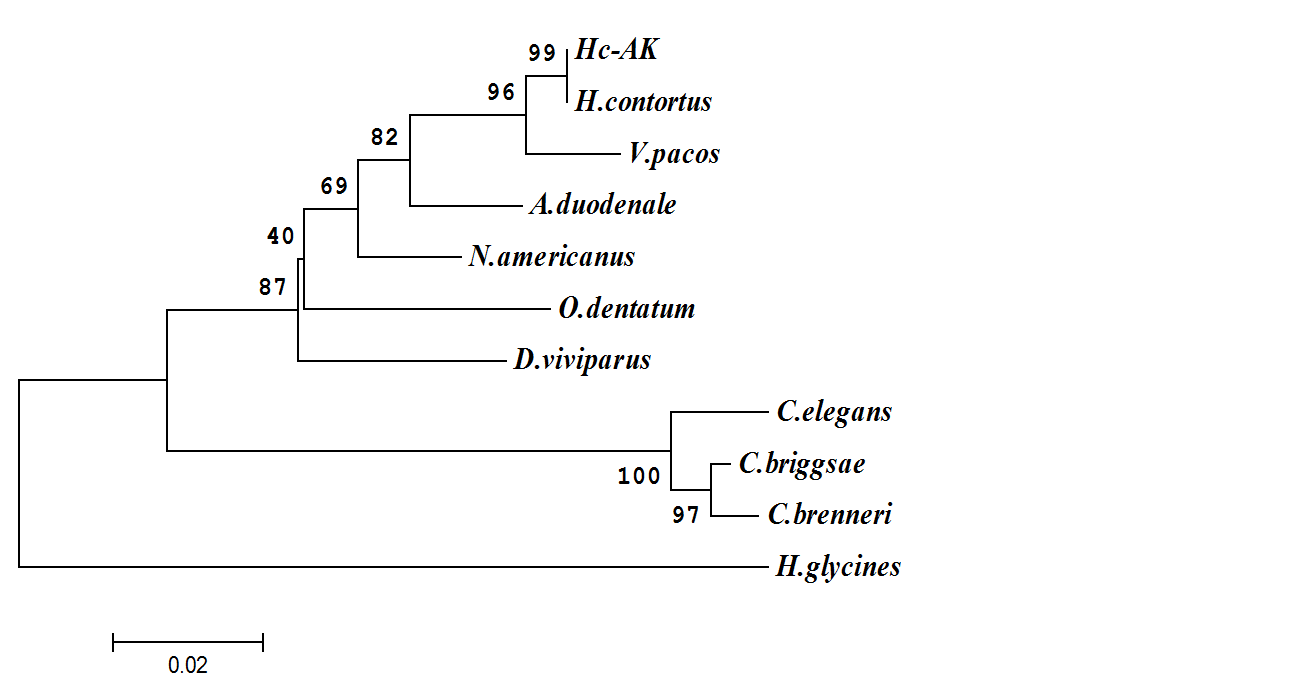


**Figure S4:** N-terminal signal peptide prediction. The amino acid sequences of Hc-AK (NCBI accession numbers: JX422018.1 was used to predict N-terminal signal peptides by SignalP 4.1 Server.


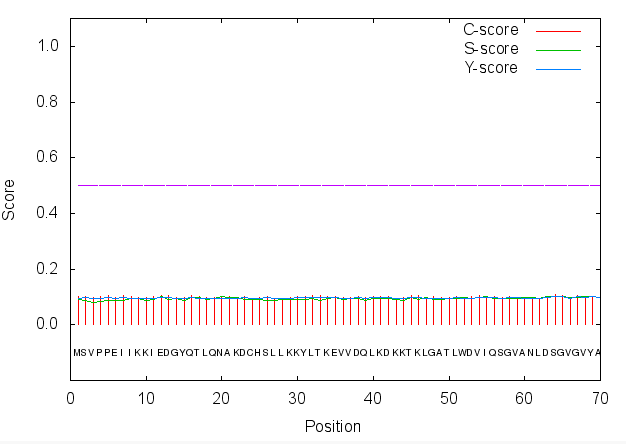


**Figure S5.** Membrane protein prediction using TMHMM Server v.2.0. The amino acid sequences of Hc-AK (NCBI accession numbers: JX422018.1) was analysed to predict transmembrane structures using TMHMM Server v.2.0. There was no transmembrane domains was predicted in this protein structure. <http://www.cbs.dtu.dk/services/TMHMM/>


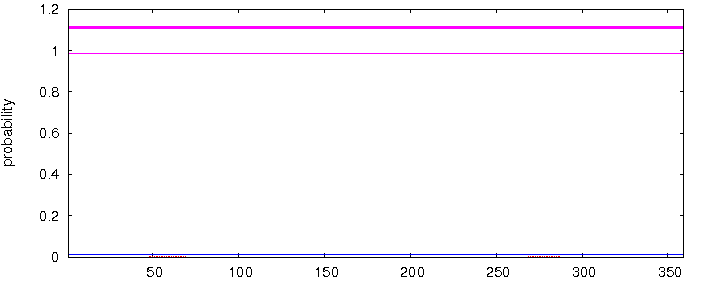


**Figure S6:** Prediction of B and T cell epitopes. Protein sequence of Hc-AK (NCBI accession numbers: JX422018.1) was used for the prediction of the B cell and T cell epitopes, that revealed 16 peptides of B cell epitopes MSV, E, DGYQTLQN, QLKDKKTK, ANLD, GVGVYAPDAEA, HNGFSPSQKQPAPDLGEGKTA, DLDPEGKYI, QGYPF, ISDPELQGTYYPLDGMTKE, YWPK, NGGNVG, APFSRD, LGTT, ISAKPDF, GEHSDSEGGVYDI, from 1-3, 6, 13-20, 39-46, 60-63, 65-75, 91-111, 115-123, 135-139, 162-180, 207-210, 239-244, 261-266, 279-282, 294-300, 316-328. And 17 T cell epitopes EIIK, KYLTKEVVD, DVIQ, GVAN, KTAQ, KYIN, KIFE, GMTK, HFLFK, RFLQ, RYWP, GIFH, RIIS, RLIK, KIIQ, GTTVR, GVYD, GLTE, EAVK, HLIE, from 6-9, 30-38, 53-56, 58-61, 109-112, 121-124, 157-160, 176-179, 189-193, 197-200, 206-209, 213,216, 233-236, 249-252, 255-258, 280- 284, 324-327, 335-338, 340-343, 351-354 were predicted.

**B cell Epitopes**


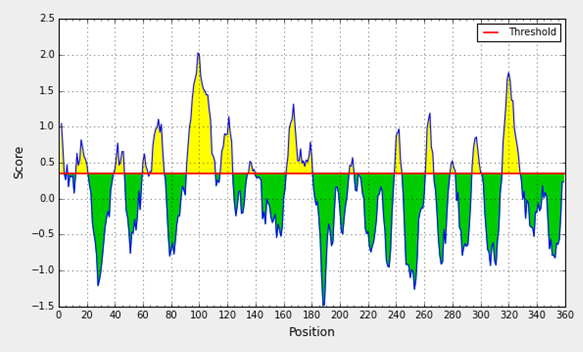


**T cell epitopes prediction**
